# Calf presence and estrous response, ovarian follicular activity and the pattern of luteinizing hormone in postpartum *Bos indicus* cows

**DOI:** 10.21451/1984-3143-AR2017-0049

**Published:** 2018-12-05

**Authors:** Eduardo Gularte Xavier, Carlos Salvador Galina, Claudio Alves Pimentel, Sandra Fiala Rechsteiner, Martin Maquivar

**Affiliations:** 1 Granja 4 Irmãos, Pelotas, RS, Brazil.; 2 Departamento de Reproducción Animal, Facultad de Medicina Veterinaria y Zootecnia,UNAM, México.; 3 Haras Santa Anita do Minuano, Capão do Leão, RS, Brazil.; 4 HISTOREP, Instituto de Biologia, UFPel, Pelotas, RS, Brazil; 5 Department of Animal Sciences. Washington State University, Pullman, Washington, USA.

**Keywords:** *Bos indicus*, LH, postpartum, temporary weaning

## Abstract

The main objective of the present experiment was to assess the effect of temporary weaning on the onset of estrus, ovarian follicular activity and secretion of luteinizing hormone in *Bos indicus* cows. Forty six mature cows were divided into three groups 1) calves were weaned for 72 h allowing auditory, olfactory and visual contact with their dams (VISUAL, n = 17), 2) calves without contact with their dams for 72 h (NC, n = 17) and 3) calves not weaned and in constant contact with their dams (CTRL, n = 12). Estrus was synchronized in all cows using CIDR for 9 days plus 2 mg of estradiol benzoate given at CIDR insertion. The VISUAL group had a greater (P *<* 0.05) proportion of cows in estrus (76.5%) compared to the CTRL group (16.7%), but no differences were found when compared to the NC group (58.8%). The VISUAL group had a greater proportion of animal that ovulated (76.5%) compared to CTRL (33.3%) and NC groups (64.7%; P = 0.059). Duration of estrus was shorter (P = 0.04) in the VISUAL (8.4 ± 4.4 hours) than in the NC (12.9 ± 13.4 hours) however, CTRL treatment (11 ± 1.4 hours) was not different from either of these two groups. LH concentration after implant removal was similar among treatments and started to increase at 36 h post CIDR removal. Only in the CTRL group was there an obvious increase by 54 h after implant removal. Follicular size increased in diameter from 36 h and were evident in treatments and control groups by 60 h (P < 0.05). The VISUAL treatment increased the number of cows ovulating and shortened the length of estrus. VISUAL and NC groups showed a similar response in follicular growth and pattern of circulating LH because of treatment.

## Introduction

Productive performance in beef cattle is closely linked to the reproductive efficiency of the herd especially during the postpartum period (Chenoweth, 1994). Resumption of ovarian activity is affected by many factors, such as forage availability, negative energy balance after parturition and the presence of the calf (Lamb *et al*., 1999; Ungerfeld *et al*., 2011). Following calving, cows experienced a period of ovarian inactivity known as postpartum anestrus, characterized by suppressed ovarian follicular growth, lack of responsiveness to gonadotropin release and absence of estrus. This phenomena is more accentuated in *Bos indicus* cattle due to the low body condition scores observed at calving, strong maternal characteristics and the deficient nutritional management during the first 50 days after calving (Baruselli *et al*., 2004; Sartori *et al*., 2010). In fact, zebu cattle shows resumption of ovarian activity around 100 days postpartum (Galina and Arthur, 1989), although recent evidence, Diaz *et al*. (2017) has shown that this timeline could be shortened. Williams *et al*. (1990) suggested that the effect of suckling prolonged the anestrus postpartum and that calf separation might hasten the onset of ovarian activity and LH secretion. In *Bos taurus* cows, the presence of a non-suckled calf delays the first ovulation postpartum (Hoffman *et al*., 1996) while Viker *et al*. (1993) demonstrated that the suckling stimuli produced by the calf prolong postpartum anestrus. Under tropical conditions, Webb *et al*. (2004) working with weaning systems reported that *Bos indicus* cows in visual but not physical contact (suckling) with their calves showed better reproductive response than those with total lack of contact with their offspring. Partial and/or complete removal of the calf can result in larger amounts of circulating gonadotropins, especially LH; this procedure demands extra resources and labor, thus limiting the use of this approach in the tropics (Abeygunawardena and Dematawewa, 2004). However, weaning for a short period of time may induce a release of gonadotropins and follicular growth and ovulation is *Bos indicus* cattle. The objectives of the present study were to evaluate the effect of temporary weaning combined with hormonal treatment on the onset of estrus and the resumption of ovarian function in *Bos indicus* cows.

## Material and Methods

All procedures involving animals and their management were approved by the Committee for the Use of Animals in Teaching and Research of the Faculty of Veterinary Medicine of the National University of Mexico.

### Location of research animals

The present study was undertaken at the Centre for Teaching, Research and Extension in Tropical Cattle of the Faculty of Veterinary Medicine, National University of Mexico, State of Veracruz, Mexico at 20° 4’N and 97° 65 3’W with a climate classified as tropical humid.

### Animals and treatments

**Fig. 1:**
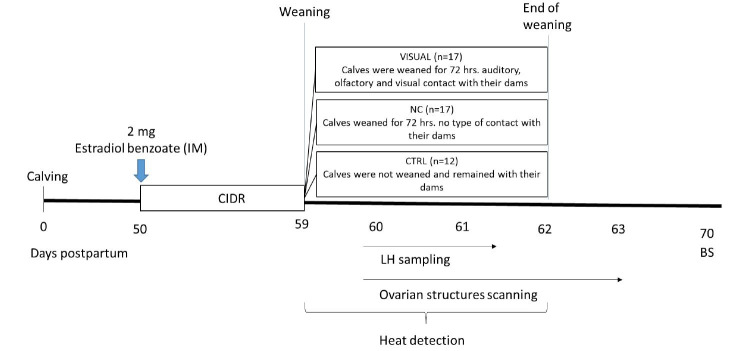
Experimental design.

Multiparous suckling Brahman (*Bos indicus*; n = 46*)* cows with an average postpartum interval of 50 days from 4 to 14 years old with a body condition score between 2.5 and 3.5 (scale 1 thin to 5 obese; Edmonson *et al*., 1989) were used in this study. Cows were randomly assigned by age, body weight and reproductive status to receive one of three treatments:

1. Calves were weaned for 72 h but in auditory, olfactory and visual contact with their dams, VISUAL, n = 17); 2) cows with no type of contact with their calves for 72 h; (NC, n = 17), and 3) control treatment where calves were not weaned and remained with their dams (CTRL, n = 12). Body weight of the cows at the onset of the study was 404.4 ± 56.1 kg (VISUAL), 415.2 ± 58.5 kg (NC) and 410.3 ± 52.3 kg (CTRL). Estrus was synchronized in all cows with use of a Controlled Intravaginal Progesterone Releasing Device (CIDR, Zoetis, Mexico) plus an intra-muscular injection of 2 mg of estradiol benzoate at implant insertion. The implants remained intravaginally for 9 days and on implant withdrawal, cows from the VISUAL and NC treatment groups were separated from their calves for 72 h as previously described (Fig. 1). All animals grazed on native pastures (*Paspalum spp*.) for the duration of the experiment.

### Sexual behavior

Cows were kept under constant visual observation for 72 h starting the day of the implant removal for detection of estrus according to the procedure recommended by Orihuela *et al*. (1983). Onset of estrus was defined as the time when the cow allowed at least three mounts in a period of 4 h or less. Estrus was defined as when three or more mounts were first received or performed by a cow with no more than 3 h between mounts (Orihuela *et al*., 1983).

### Reproductive evaluations

Serum progesterone concentration determined that 44% of cows were cycling at the beginning of the experiment; these animals were distributed equally among treatments. Ovarian evaluations were performed using an Aloka model SSD – 500 with a linear transductor probe of 7.5 MHz. All cows were tested by ultrasound for the presence of ovarian follicles every 6 h starting 24 h (Day 60) after implant removal until 102 h (day 63) or until ovulation occurred. Ovulation was defined as the time when a follicle larger than 8 mm in diameter was no longer found to be present in the ovary.

Ovulation was validated by the presence of a functional corpus luteum (CL) 7 days after (day 70) the last ultrasound and substantiated by progesterone concentrations greater than 1 ng/ml.

### Blood sampling and hormone analysis

Blood samples were collected during the ultrasound evaluations and also twice a week for 4 weeks before the start of hormonal treatment to establish the percentage of females in anestrus. Other samples were taken to measure progesterone concentration at the time of implant removal and 7 days later after calves were reunited with their dam. Additionally, following CIDR withdrawal/weaning of the calves, all cows underwent intensive blood sampling for LH determination (from 24 to 66 hours) along with ultrasound examination of the ovaries. Blood samples were taken by tail vein or artery puncture, centrifuged at 1500 rpm for 10 minutes, and kept frozen until liquid radioimmunoassay (RIA) specific for bovine LH with 120 h of incubation at 4°C. USDA-bLH radiolabeled [I125] as marker using iodine IODO-GEN technique (Perera-Marin et al., 2005). The intra-assay coefficient of variation was 7.8% (1.8 ng/tube) and the assay sensitivity was 0.02 ng/tube. All samples were analyzed in one duplicate assay. Plasma progesterone concentration was analyzed in duplicate using a commercial radioimmunoassay analysis (RIA) kit (Coat-a-Count, Siemens Medical Solutions Diagnostics, Los Angeles, CA, USA). Coefficient of variation for the assay was 4.1% and sensitivity was 0.1ng/ml.

### Statistical analysis

Variables measured were implant withdrawal/weaning to onset of estrus and end of estrus, time of ovulation and time of LH increase, as well as onset of estrus to ovulation, end of estrus to ovulation and time of LH increase to ovulation. Variables were analyzed using a general lineal model, the GLM procedure in the SAS statistical software (2004). LH concentration and dominant follicle diameter were analyzed by ANOVA PROC MIXED procedure in SAS, using time as a repeat measure. An LH surge was defined as an increase in LH concentration of at least two standard deviations above the individual baseline observed during the first 48 h of intensive bleeding (Mikeska and Williams, 1988). Cows that had ovulated and shown estrus by a Chi-square test.

## Results

No statistical differences were observed in estrous response after implant withdrawal between cows from the VISUAL (76.5%) and NC (58.8%) treatment groups. However, cows in the VISUAL group had a greater (P < 0.05) estrous response compared to CTRL treatment (16.7%). No differences were detected among cows of the NC and CTRL groups. Furthermore, the VISUAL group tended to have a greater percentage of animals that ovulated (76.5%) compared to the NC treatment (64.7%) and control group (33.3%) (P = 0.059). Females from the VISUAL group had a shorter estrus (8.4 ± 4.4 hours) than the NC group (12.9 ± 13.4 hours; P = 0.04). However, no differences among the VISUAL and NC groups were seen when compared with the CTRL group (11 ± 1.4 hours).

Overall, estrous response to treatment, regardless of weaning status, was 54.3%, and overall ovulation rate was 60.8%. Onset of estrus for all treatments was around 35 h after implant withdrawal (Table 1). Animals from the VISUAL group had ovulated by 67 h after weaning/implant withdrawal and cows from the NC and CTRL groups around 72 h, this difference was not significant. Groups showed a similar pattern of increases in LH concentration; the VISUAL group at 42 h, the NC and CTRL groups around 47 h. Interestingly, in the CTRL group from the onset of estrus to ovulation, only one animal that exhibited estrus ovulated, whereas the remaining animals (n = 3) that ovulated did not show overt signs of estrus.

**Table 1:** Effect of the presence or absence of the calf after an estrous synchronization program on reproductive variables in postpartum *Bos indicus* cows.

Variables (h)		VISUAL n = 17	NC n = 17	CTRL n = 12
Implant withdrawal/ weaning to:	Onset of estrus	34.9 + 6.4	34.8 + 6.2	36.5 + 10.6
	End of estrus	42.3 + 5.5	46.7 + 4.2	46.5 + 9.2
	Ovulation	67.4 + 11.3	72.0 + 14.9	73.5 + 12.4
	LH increase	42.5 + 8.2	47.4 + 11.8	48.0 + 8.5
Onset of estrus to ovulation		32.7 + 8.9	34.3 + 10.7	31.0*
End of estrus to ovulation		24.7 + 10.7	22.2 + 10.8	20.0*
LH increase to ovulation		24.9 + 8.0	24.5 + 8.6	25.5 + 5.7

VISUAL = calves partially removed; NC = calves completely removed; CTRL= calves were not removed; Variables are expressed in means ± SD; *Values represent one observation of a cow that displayed estrous behavior and had an ovulation.

**Fig. 2:**
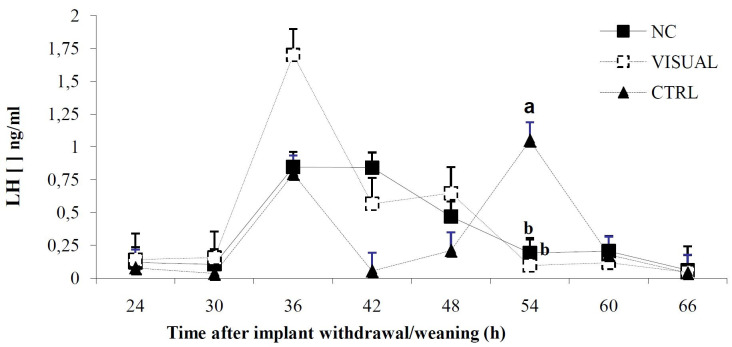
Effect of the presence or absence of the calf after an estrous synchronization program on luteinizing hormone in postpartum *Bos indicus* cows; VISUAL = calves partially removed; NC = calves completely removed; CTRL = calves were not removed;. Different superscripts among time points denotes a difference (P < 0.05).

LH concentration started to increase at 36 h in the three groups. However, only in the CTRL group was there any evident increase in LH by 54 h after implant removal compared to cows in the VISUAL and NC groups (Fig. 2).

**Fig. 3:**
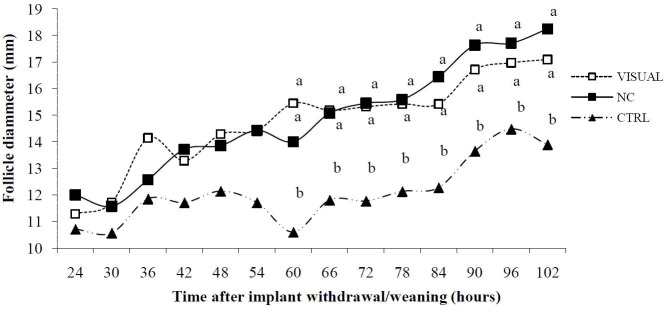
Effect of the presence or absence of the calf after an estrous synchronization program on dominant follicle diameter in postpartum *Bos indicus* cows; VISUAL = calves partially removed; NC = calves completely removed; CTRL= calves were not removed; Different superscripts among time points denotes a difference (P < 0.05).

Additionally, LH increase for all treatments occurred around 25 h (Table 1). Ovulation occurred around 22 h after cessation of estrus. In terms of follicular growth 60 h from implant withdrawal, the diameter of dominant follicles of cows in the VISUAL and NC groups were larger (P < 0.05) than those of cows from the CTRL group (Fig. 3).

## Discussion

Many factors influence the resumption of ovarian activity postpartum such as nutrition, environmental age and genetics (Jolly *et al*., 1995). One of the most important factors is the effect of suckling. Cows that were nursing calves delayed the resumption of ovarian follicular growth, ovulation and establishment of a normal lifespan CL (Abeygunawardena and Dematawewa, 2004). Reducing the time that the cow nurses a calf by partial or total weaning procedures increases the number of animals that resume ovarian activity and ovulate (Perea *et al*., 2008; Pinheiro *et al*., 2009; Perez-Torres *et al*., 2015).

Rhodes *et al*. (2003) reported that treating anestrous cows with progestagen plus estradiol induces estrus response and ovulation in almost 90% of the cows. In contrast, only 54% of the cows exhibited estrus and 60% had ovulations in the present study. Findings more consistent with a previously reported 50% response rate in Zebu-type cattle (Vasconcelos *et al*., 2009; Sá Filho et al., 2009; Hernandez *et al*., 2015). Incidence of estrus was enhanced by weaning. The percentage of cows in estrus in the present study was twice that of cows in the control treatment (cows that had a synchronized time of estrus and their offspring not removed). This result is consistent with previous evidence that calf separation has a dramatic effect on estrous expression regardless of the method used to separate the calves (Williams *et al*., 1996; Quintans *et al*., 2009, Quintans *et al*., 2010).

Cows with calf contact had a greater tendency to display overt signs of estrus (76.5%) than those with no calf contact (58.8%). A similar tendency was observed in the proportion of ovulating cows (76.0% compared with 64.0%, respectively). Comparable results were previously observed (Webb *et al*., 2004) where 65% of animals subjected to partial weaning of their calves ovulated after the estrous synchronization program was imposed. Progestagen is another factor that could improve ovulation rates; however, its effectiveness is reported to be variable, ranging between a success rate of between 5 and 70% to induce estrus and ovulation (Day, 2004; Perez-Torres *et al*., 2015). In the present study, regardless of the separation method used, an average of 60% of dams ovulated.

Earlier studies demonstrated that GnRH release is suppressed in suckling beef cows; consequently the episodic release of LH is inhibited affecting ovarian follicular growth (Yavas and Walton, 2000). The recovery of the episodic secretion pattern has been reported to occur between 15 and 30 days postpartum. LH concentration increased around 36 h after implant withdrawal/weaning in all groups. However, in the CTRL group there was a statistically different LH concentration at 54 h probably due to the effect of calf separation together with the hormonal treatment, favoring GnRH release followed by LH secretion. In the case of the CTRL animals, increase in LH was probably induced by the hormonal treatment alone.

Results of the present study are consistent with those of Maquivar *et al*. (2007) who found an increase in LH occurring in the lead-up to ovulation. This increase occurred around 24 h in estrous cycling *Bos indicus* cows synchronized with use of a progestagen. Regardless of the weaning treatment, cows ovulated 25 h after the pre-ovulatory surge of LH release. Interestingly, dominant follicular diameter was greater in the weaning treatments than in the CTRL group starting at 60 h post implant withdrawal/weaning. Similar reports have been published recently (Mondragon et al., 2016). This trend continued until the end of the ultrasonic assessments in the present study. Our results suggest agreement with previous observations that cows with smaller follicles have less opportunity to become pregnant (Sá Filho *et al*., 2009).

Weaning combined with the use of hormonal treatment was shown in the present study to be effective for inducing estrus and establishing estrous cycles, confirming earlier studies that weaned cows have a tendency to an earlier onset of estrous cycles, thus increasing the probability of becoming pregnant at the onset of the breeding season. Further research is necessary on the impact of the weaning procedure on cortisol levels and/or stress that may impede the onset of estrus and/or ovulation. Pérez et al. (2017) reported minor differences in cortisol levels between treatments and control. However, in-depth study on the effect of weaning on specific metabolic parameters such as adipose tissue mobilization and estradiol concentration is needed.

Results from the present experiment indicate that calf removal but allowing contact with dams increased the number of ovulating cows and shortened the time to the initiation of estrous cycles following calving. Both treatments (VISUAL and NC) induced a more homogeneous response in ovarian follicular growth and LH pattern compared with cows from the CTRL group.
